# Optimization studies of BTX removal by magnetite coated oleic acid obtained from microwave-assisted synthesis using response surface methodology

**DOI:** 10.1038/s41598-022-22716-w

**Published:** 2022-11-03

**Authors:** Makhosazana Masuku, Linda Ouma, Saheed Sanni, Agnes Pholosi

**Affiliations:** 1 Biosorption and Water Treatment Research Laboratory, Vaal University of Technology, Private Bag X021, Vanderbijlpark, 1900 South Africa; 2 Department of Science, Technology and Engineering, Kibabii University, P. O. Box 1699, Bungoma, 50200 Kenya

**Keywords:** Chemistry, Engineering, Materials science, Nanoscience and technology

## Abstract

Benzene, toluene and xylene (BTX) are volatile organic compounds released into the environment, that require urgent removal to avoid adverse health effects. In this work, the modelling and optimization of the preparation factors for magnetite coated oleic acid (MNP-OA) composite from microwave synthesis using response surface methodology were conducted to maximize BTX removal, and iron content. The influence of five crucial preparation variables: the Fe^3+/^Fe^2+^ solution volumes, microwave power, volume of ammonia water (VAW), reaction time and volume of oleic acid (VOA) on the iron content (% Fe), and BTX adsorption capacity were investigated. The analysis of variance results revealed that VOA and VAW were the most influential factors for high % Fe content, and improved BTX removal. The % Fe, and BTX adsorption capacity for MNP-OA composite at optimized experimental conditions were estimated to be 85.57%, 90.02 mg/g (benzene), 90.07 mg/g (toluene), and 96.31 mg/g (xylene).

## Introduction

In general, the surrounding environment especially the waterways comprise of emerging volatile organic compounds (VOCs) generated from different industries and human activities. These VOCs can cause serious carcinogenic, mutagenic and teratogenic effects in human health and ecosystem after long term bioaccumulation, hence there is an urgent need to remove these VOCs from the environment, for human health protection^1–3^. Among these VOCs, benzene, toluene, xylene (BTX) are priority hydrophobic pollutants generated from gasoline, petroleum, and oil derivatives^4^. Human health hazard from BTX compounds at low concentration include cancerous cells, headache, damage to the human nervous system, dizziness, and even death at advanced stages^5^. Several approaches such as membrane technology, advanced oxidation processes, and adsorption, have been utilized for the removal of BTX compounds in the environment, many of which involve high costs, harmful side effects and conversion to other substrate after treatment^2,5,6^. However, adsorption technology is a versatile, and cost effective method for the removal of VOCs, due to its ease of operation, high efficiency, and additional value in the recovery of the adsorbate and target VOCs for reuse^4^.

The application of magnetic nanosorbents comprising of Hematite (Fe_2_O_3_), Ferrous oxide (FeO), maghemite (γ-Fe_2_O_3_) and magnetite (Fe_3_O_4_) have been proven to be effective in detoxifying a wide variety of VOCs through adsorption process. From aforementioned, magnetite nanoparticles (MNPs) have great potential in the removal of VOCs in water due to its ease of synthesis, excellent physical, and chemical properties^7–9^. Some of the notable properties of MNPs in wastewater treatment include low-cost, biocompatibility, high surface area to volume ratio for adsorption due to high active sites, less toxicity, and superparamagnetic behavior^7,10–12^. The MNPs morphology, and growth size can easily be controlled through the synthetic approach, and separated from the solution after treatment by a magnetic field without the use of expensive and energy-intensive processes^13,14^. Bare MNPs however, tend to agglomerate which results in deterioration of their magnetic properties thus limiting their potential applications^15–18^*.* As such, a proper surfactant and synthetic approach is required for controllable growth and stability of MNPs. One essential organic surfactant that can coat magnetite to reduce agglomeration is oleic acid. The polar head group on the oleic acid is attached on the magnetite surface and the long non-polar hydrophobic alkyl tail extends into the solution causing magnetite to be hydrophobic and dispersible in organic solvents, thus providing steric stability to the magnetite nanoparticles^19,20^.

A host of different methods comprising of thermal decomposition, co-precipitation, solvothermal, sonolysis, hydrothermal, microemulsions, and microwave-assisted synthesis (MAS) have been utilized for MNPs synthesis^8,21–23^. The MAS is a promising synthetic technique among the above-mentioned methods because of its capability to produce MNPs with narrow size distributions^8,23^. This is ascribed to the efficient heating to the desired temperature, high phase purity, increased reaction kinetics, reduced synthesis time, easy functionalization, high crystallinity, and reproducibility^24–28^. However, the limiting factor associated with MNPs is the optimization of process variables in the formation of MNPs with high iron content required for magnetic functionality in the chemical composition of the nanocomposite, ease of separation desirable for adsorption process^20,29^, and other applications^30^.

Studies from previous work revealed that the volume of Fe^3+^/Fe^2+^solution, microwave power (MP), volume of ammonium water (VAW), reaction time, and volume of oleic acid (VOA) as surfactant play huge role in growth distribution, stability and physicochemical properties of MNPs with regards to their potential applications^8,31,32^. However, these works have not fully described how simultaneous optimization of these aforementioned parameters may influence the efficient application of magnetite coated with oleic acid (MNP-OA) for benzene adsorption capacity (BAC), toluene adsorption capacity (TAC) and xylene adsorption capacity (XAC). Their influence on the iron content (% Fe) required for efficient magnetic separation after adsorption process has also not been reported. Thus, the simultaneous optimization of these synthesis parameters is desirable to obtain high adsorption activities for the MNP-OA sorbent material on the BTX pollutant removal, and promotes effective recovery.

Response surface methodology (RSM) is an efficient statistical method utilized by researchers for modeling, and simultaneous optimization of various preparation variables rather than one factor at a time (OFAT), in predicting the best optimum conditions with a minimum number of experiments, hence minimizing the possible errors, synthesis time and cost associated with OFAT approaches^33,34^. To the best of our knowledge, the preparation of MNP-OA from MAS employing RSM approach in maximizing the % Fe content, BAC, TAC, and XAC removal has not been studied before. Also, the role of these synthesis variables cannot be overlooked, considering their relevance in adsorption mechanism, hence this present study further validates the influence of variables (most significant from RSM model) on the MNP-OA composite attributes on BTX removal.

Therefore, the present study focuses on modelling, and optimization of MNP-OA composite preparation parameters from MAS, in achieving maximum % Fe content, and enhanced BTX removal. Through the fitting of the regression model from the analysis of variance, the interactive influence of molar volume of Fe^3+^/Fe^2+^ solution, MP, VAW, time, and VOA significant effect on % Fe content and enhanced BAC, TAC, and XAC removal were also analyzed by the response surface plots in this study. The importance of this present study is not only in successful development of MNP-OA adsorbent using RSM model, but also on the exploitation of the model, in understanding the most significant variable influence on the physicochemical attributes of MNP-OA composite on enhanced adsorption of BTX.

## Materials and methods

### Materials

Sulphuric acid (H_2_SO_4_ > 98%), Ferrous sulphate hydrate (FeSO_4_·7H_2_O > 98%) Ferric chloride hydrate (FeCl_3_.6H_2_O > 99%) and Hydrochloric acid (25%), were provided by Merck. Ammonium hydroxide (NH_4_OH, 25%) was supplied by Labchem (South Africa). Toluene, 99.8%, Xylene, 99.0%, Benzene, ≥ 99.9%, Ethanol, 96%, and Oleic acid (OA) ≥ 99% were all purchased from Sigma-Aldrich. All chemicals were used without any further purification.

### Procedures

#### Synthesis of the magnetite-oleic acid composites via microwave method

A stock solution of 0.2 M FeCl_3_·6H_2_O and 0.1 M FeSO_4_·7H_2_O was freshly prepared in an acidic medium of HCl, and H_2_SO_4_ respectively. The synthesis of the magnetite-oleic acid (MNP-OA) composite was carried out in a three-neck flask through mixing 50 mL of FeCl_3_·6H_2_O and 25 mL FeSO_4_.7H_2_O solution, 100 mL ethanol solution (40% v/v) and ammonium hydroxide solution (25%) at different volumes (2, 5, 8 mL) was used as a precipitating agent. The stirred solution was further purged with nitrogen gas, heated at 70 °C under constant magnetic stirring at 300 rpm for 10 min. A black precipitate solution was formed after 10 min, thus oleic acid at different volume (0.2, 2.1, 4 mL) was added to this solution to introduce hydrophobicity, as the solution was left to continue stirring at different times (5, 10, 15 min) after the oleic acid addition. The stirred solution was then subjected to microwave assisted heating (model Kelvinator KML45B, maximum power of 1100 W, frequency of 2450 MHz) for another 5 min at different microwave power (200, 500, 800 W). The black MNP-OA precipitate formed was later separated by magnetic decantation. The precipitate was washed several times with distilled water to remove any impurities present then washed with ethanol, dried at 60 °C for 2 h, and stored in the dark for further analysis. The magnetite (MNP) material was prepared above without addition of oleic acid. The description of the batch adsorption studies for BAC, TAC and XAC removal by MNP-OA composite is found in the supplementary information S1.

#### Characterization

X-ray diffraction (XRD) analysis was conducted to identify the crystallographic structure of the MNP-OA and MNP composite using an X'Pert PRO (Japan) X-ray diffractometer (CuKα anode; π ≈ 0.154 nm). Particle diameters were calculated following Debye–Scherrer's equation to confirm the formation of nano-sized particles. The particle morphology, size and structure of MNP and MNP-OA were determined on a FEI Tecnai F20 Transmission electron microscope (TEM) at an acceleration voltage of 200 kV High-Resolution images of the materials for shape and size measurements were obtained. The magnetic properties of the optimized adsorbents were carried out on a Lake Shore model 735 vibrating sample magnetometer at room temperature. Moreover, the Fourier transform infrared (FTIR) spectroscopy of MNP, and MNP-OA adsorbents were recorded within the range of 500–4000 cm^−1^ using PerkinElmer spectrum 400.

#### Experimental design and RSM optimization

The Design Expert Software (Version 11.1.2), from Stat-ease Inc., Minneapolis, MN, USA. was adopted to study the data analysis and statistical design of experiments^33,35^. In the present study, the response surface methodology (RSM) using Central Composite Design, (CCD) approach was applied to optimize the preparation of the magnetite-oleic acid composite for the adsorption of BTX compound from aqueous solution, and to assess the relationship among five independent variables: molar volume of Fe^3+^/Fe^2+^ solution (*A*), microwave power (*B*), volume of ammonium water (VAW) (*C*), time (min), and volume of oleic acid (*E*). The four responses of the study comprises % Fe content and adsorption capacity (mg/g) of BTX, as the results are shown in Table 1. The variables were chosen in accordance with our previous studies^20^. The independent variables used were varied between the lower (− 1) and the higher (+ 1) levels. The adsorption capacity of BTX from the RSM model (Table 1) comprise of 42 randomized runs including 8 replicates. The model equation of response (Y) of five independent variables was given in the following equation below:

1 $$ Y = b_{0} + \sum\limits_{i = 1}^{5} {b_{i} x_{i} + \sum\limits_{i = 1}^{5} {b_{ii} x_{i}^{2} + \sum\limits_{i = 1}^{5} {\sum\limits_{j = i1}^{5} {b_{ij} x_{i} x_{j} } } } } $$

where Y is the predicted response for % Fe content, BAC, TAC, and XAC respectively, xi, and xj are the independent variables in coded levels, b_i_, b_ii_, b_ij_ are the coefficient for the linear, quadratic, and interaction effect, respectively, and b0 is the model coefficient.

**Table 1:** Variable levels for RSM experiment.

Variables			Levels				
Factor	Name	Units	Minimum	Maximum		Values of code	
A	Fe^3+^/Fe^2+^	mL	0.50	2.0	− 1.0 = 0.5	1.0 = 2.0	0 = 1.25
B	Microwave Power	W	200	800	− 1.0 = 200	1.0 = 800	0 = 500
C	Volume of NH_4_OH	mL	2.0	8.00	− 1.0 = 2.0	1.0 = 8.0	0 = 5.0
D	Time	Min	5.0	15.00	− 1.0 = 5.0	1.0 = 15.0	0 = 10.0
E	Volume of Acid	mL	0.2	4.00	− 1.0 = 0.2	1.0 = 4.0	0 = 2.10

RSM, response surface methodology; Fe^3+^/Fe^2+^, iron (III)/iron (II); NH_4_OH, ammonium hydroxide.

## Results and discussion

### Development of regression model equation and statistical analysis

In this experimental design, a total of 50 experimental runs were performed and the results are presented in Table 2. The observed percentage iron content (% Fe) for the MNP-OA composite ranged between 49.79 to 85.95%. The adsorption capacity ranged between 0.5 to 116 mg/g for benzene, 1.0 to 82 mg/g, for toluene and from 10 to 89 mg/g for xylene as presented in Table 2. Central composite design (CCD) was used to develop a relationship between the MNP-OA composite preparation variables influence on the % Fe content, BAC, TAC, and XAC respectively. Based on the model analysis, the final regression models, in terms of their coded factors, excluding the insignificant terms, are expressed by the second-order polynomial below:

2 $$ \begin{aligned} \% \,{\text{Fe}} & = 78.02 + 2.25A + 1.66B + 0.49C - 6.67E - 5.08AD \\ & \quad + \,0.96AE + 1.38BC - 1.09BD + 0.92BE - 1.90CD \\ & \quad - \,2.70CE - 2.70DE + 4.24B^{2} - 9.66D^{2} - 4.71E^{{2}{}} \\ \end{aligned} $$

3 $$ \begin{aligned} {\text{BAC}} & = 63.20 - 4.18B - 4.43C + 1.56D - 3.81E + 3.24AB - 1.24AC \\ & \quad + \,2.19AE + 5.02BC - 3.20BD - 2.66CD + 3.87CE + 2.32DE \\ & \quad - \,12.45A^{2} + 18.08B^{2} - 27.63C^{2} - 17.17D^{{2}{}} + 16.52E^{2} \\ \end{aligned} $$

4 $$ \begin{aligned} {\text{TAC}} & = 65.94 - 3.71B - 4.57C + 1.78D - 4.17E + 3.07AB + 2.52AE \\ & \quad + \,5.85BC - 3.40BD - 2.43CD + 3.48CE + 1.96DE \\ & \quad - \,12.76A^{2} + 17.53B^{2} - 28.31C^{2} - 16.30D^{2} + 16.19E^{2} \\ \end{aligned} $$

5 $$ \begin{aligned} {\text{XAC}} & = 70.22 - 4.25B - 4.53C + 1.52D - 3.64E + 3.23AB - 1.39AC \\ & \quad + \,2.20AE + 5.03BC - 3.12BD - 2.67CD + 4.06CE + 2.27DE \\ & \quad - 7.18A^{2} + 23.91B^{2} - 46.76C^{2} - 11.54D^{2} + 21.76E^{2} \\ \end{aligned} $$

**Table 2:** Experimental design and results for preparation of Oleic Acid Coated Magnetite with % Fe content and adsorption capacities for BTX.

Variables						Responses			
Runs	Fe^3+/^Fe^2+^ (mL)	Microwave power (W)	NH_4_OH volume (mL)	Time (min)	Volume of oleic acid (mL)	% Fe	Benzene adsorption capacity (mg/g)	Toluene adsorption capacity (mg/g)	Xylene Adsorption capacity (mg/g)
1	1.25	500.00	2.00	10.0	2.10	77.4	37.56	39.88	27.35
2	1.25	500.00	5.00	10.0	2.10	78.9	62.616	65.67	72.33
3	2.00	800.00	2.00	15.0	4.00	58.7	39.656	40.01	49.67
4	0.50	200.00	2.00	5.00	0.20	58.5	62.074	64.45	72.221
5	2.00	800.00	8.00	15.0	0.20	76.7	30.78	34.13	40.18
6	2.00	800.00	2.00	5.00	4.00	73.1	25.78	29.66	35.75
7	0.50	800.00	2.00	5.00	4.00	55.1	22.88	26.45	32.55
8	1.25	500.00	8.00	10.0	2.10	77.2	33.7	35.67	13.9
9	1.25	500.00	5.00	10.0	4.00	69.1	77.67	76.9	87.17
10	0.50	200.00	2.00	5.00	4.00	50.6	34.158	36.12	44.321
11	2.00	800.00	8.00	15.0	4.00	55.1	37.148	40.78	47.567
12	0.50	500.00	5.00	10.0	2.10	77.9	51.842	56.43	61.341
13	0.50	800.00	2.00	15.0	4.00	65.4	21.764	24.65	31.123
14	1.25	200.00	5.00	10.0	2.10	82.2	87.002	89.55	97.26
15	1.25	500.00	5.00	10.0	2.10	80.8	62.47	66.67	72.47
16	1.25	500.00	5.00	10.0	2.10	78.6	60.99	62.12	70.99
17	1.25	500.00	5.00	10.0	2.10	76	67.398	68.45	77.567
18	0.50	200.00	8.00	15.0	4.00	52.4	45.13	48.56	55.63
19	0.50	200.00	2.00	15.0	0.20	80	71.406	76.33	81.06
20	1.25	500.00	5.00	10.0	2.10	75.3	61.664	64.34	71.264
21	2.00	800.00	2.00	5.00	0.20	70.3	46.35	47.88	56.45
22	1.25	500.00	5.00	10.0	2.10	77.7	61.55	65.09	71.85
23	0.50	200.00	2.00	15.0	4.00	64.7	57.542	54.66	67.52
24	0.50	200.00	8.00	15.0	0.20	79.5	37.634	38.78	47.564
25	2.00	200.00	8.00	15.0	4.00	50.3	37.284	36.45	47.684
26	0.50	800.00	8.00	15.0	4.00	58.7	31.036	32.11	41.6
27	2.00	200.00	8.00	5.00	4.00	64.9	28.084	27.89	38.84
28	0.50	200.00	8.00	5.00	4.00	46.4	34.1	34.23	44.98
29	2.00	200.00	8.00	5.00	0.20	76.8	24.42	24.45	34.42
30	2.00	200.00	2.00	5.00	4.00	69.2	42.47	44.8	52.17
31	1.25	500.00	5.00	15.00	2.10	69.3	47.688	49.78	57.234
32	2.00	200.00	2.00	15.00	4.00	58.3	54.486	59.23	64.21
33	2.00	800.00	8.00	5.00	4.00	74.4	42.358	43.23	52.564
34	2.00	800.00	2.00	15.00	0.20	68.9	47.276	44.23	57.176
35	1.25	500.00	5.00	5.00	2.10	69.8	44.486	49.78	54.46
36	0.50	200.00	8.00	5.00	0.20	65.9	44.1	42.12	54.1
37	2.00	200.00	2.00	5.00	0.20	69.6	49.542	50.55	59.52
38	0.50	800.00	2.00	15.00	0.20	80.1	40.478	46.23	50.478
39	2.00	200.00	2.00	15.00	0.20	74.1	62.1	65.34	72.1
40	0.50	800.00	8.00	5.00	4.00	58.1	32.099	32.48	43.12
41	2.00	200.00	8.00	15.00	0.20	75.1	29.41	29.87	39.11
42	1.25	800.00	5.00	10.00	2.10	84.7	75.672	77.68	85.342
43	1.25	500.00	5.00	10.00	2.10	80.6	63.398	65.31	73.564
44	0.50	800.00	8.00	15.00	0.20	80.4	30.35	35.67	40.95
45	0.50	800.00	2.00	5.00	0.20	58.3	43.946	41.66	53.123
46	1.25	500.00	5.00	10.00	2.10	78.9	64.98	68.76	74.34
47	2.00	500.00	5.00	10.00	2.10	80	49.77	50.22	59.07
48	1.25	500.00	5.00	10.00	0.20	79.9	81.888	87.65	71.126
49	0.50	800.00	8.00	5.00	0.20	72	46.368	49.78	56.458
50	2.00	800.00	8.00	5.00	0.20	85.1	44.908	50.71	54.143

**Figure 1:**
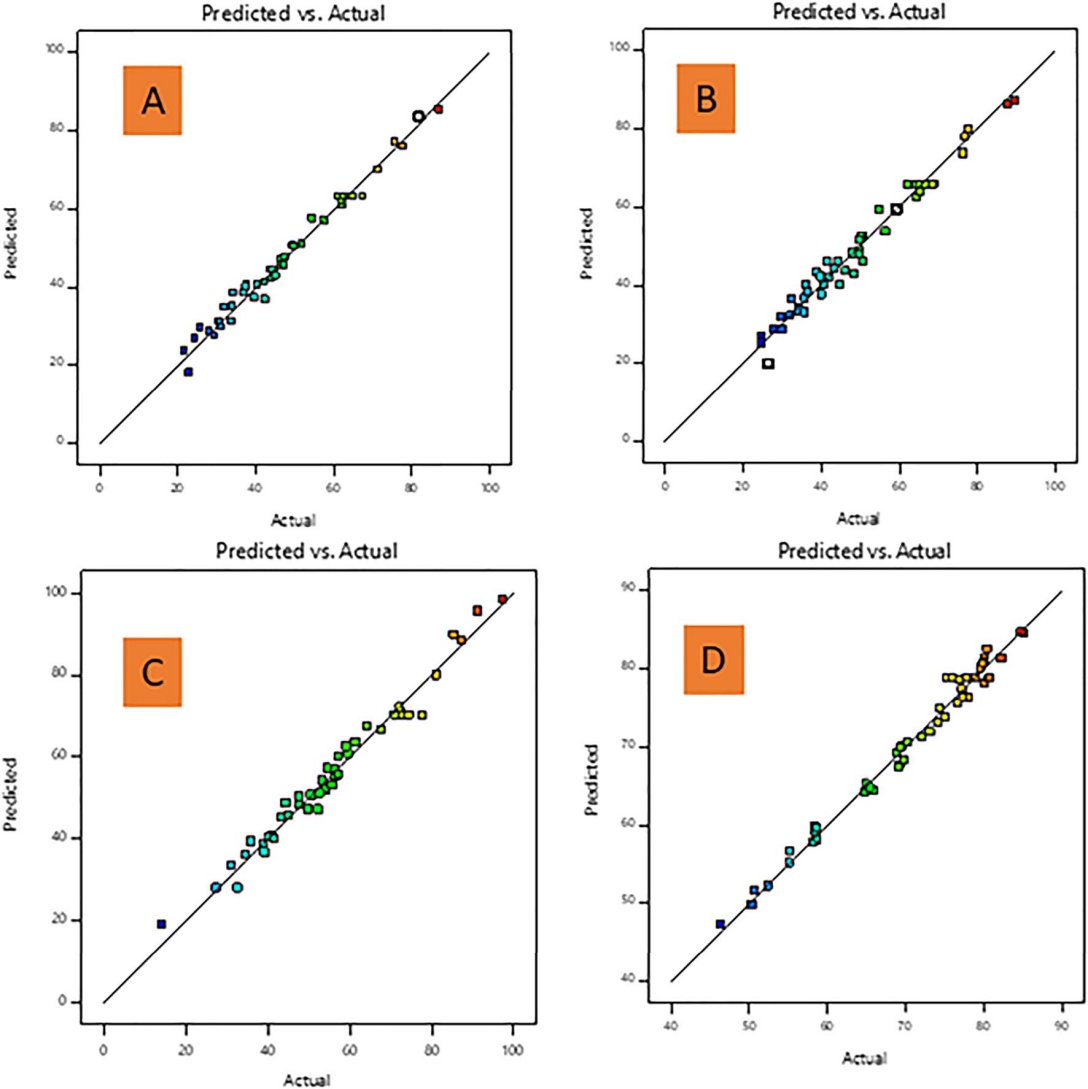
Plot of actual values against predicted values of responses for (**A**) % Fe content of magnetite-oleic acid (MNP-OA) composite, (**B**) benzene, (**C**) toluene, and (**D**) xylene adsorption capacity.

The positive sign in front of the terms indicates a synergistic effect, whereas a negative sign indicates an antagonistic effect^33,36^. Table S1–S4 presented the analysis of variance (ANOVA) of regression of the predicted response from the quadratic models for % Fe content, BAC, TAC and XAC. The calculated F values from the ANOVA (Table S1–S4) for the % Fe content, BAC, TAC and XAC were found to be 96.81, 85.36, 55.63, 61.42 respectively, with lower probability (< 0.0001) indicating that the models were significant. Values presented in Table S1–S4 with “Prob > F” less than 0.05 indicates model terms are significant, whilst the insignificant terms are omitted from the tables. The calculated coefficient of determination (R^2^) values were 0. 9852, 0.9833, 0.9746, and 0.9769 for the % Fe content, BAC, TAC and XAC respectively which highlights an acceptable accuracy of the datas with the model. The adequacy of the model equation in describing the experimental data was validated from the plot of predicted versus actual values. The relationship between predicted and actual values of % Fe content and BAC, TAC and XAC (mg/g) are indicated in Fig. 1A,D. It is observed that the plots show a high correlation between the predicted and actual results indicating, this model can be used to navigate the design space.

### Effect of preparation variables on % Fe content and BTX adsorption capacity

**Figure 2:**
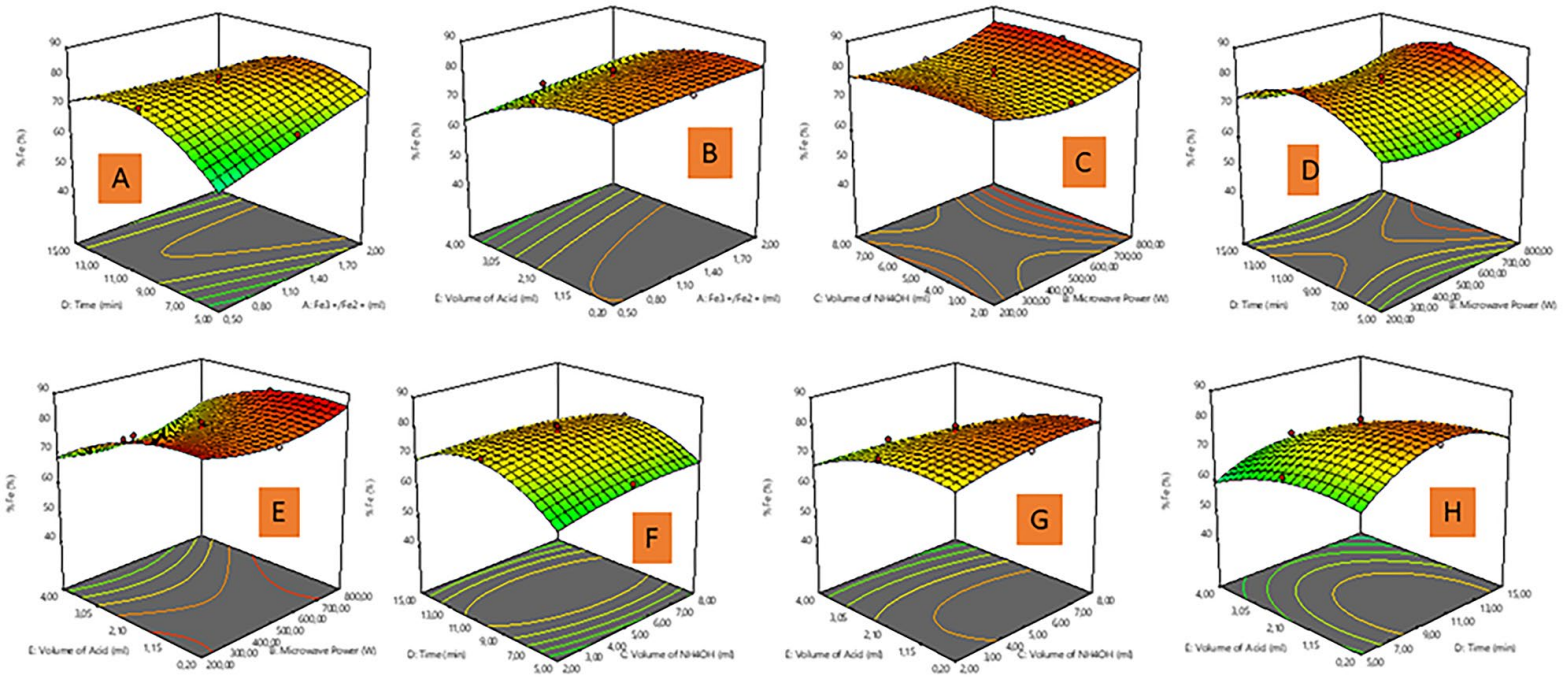
3D response plots of (**A**) Fe^3+/^Fe^2+^ solution and time, (**B**) Fe^3+/^Fe^2+^ solution and volume of oleic acid, (**C**) microwave power and volume of ammonium hydroxide, (**D**) microwave power and time, (**E**) microwave power and volume of oleic acid, (**F**) volume of ammonium hydroxide and time, (**G**) volume of ammonium hydroxide and volume of oleic acid, (**H**) time and volume of oleic acid on % Fe content.

**Figure 3:**
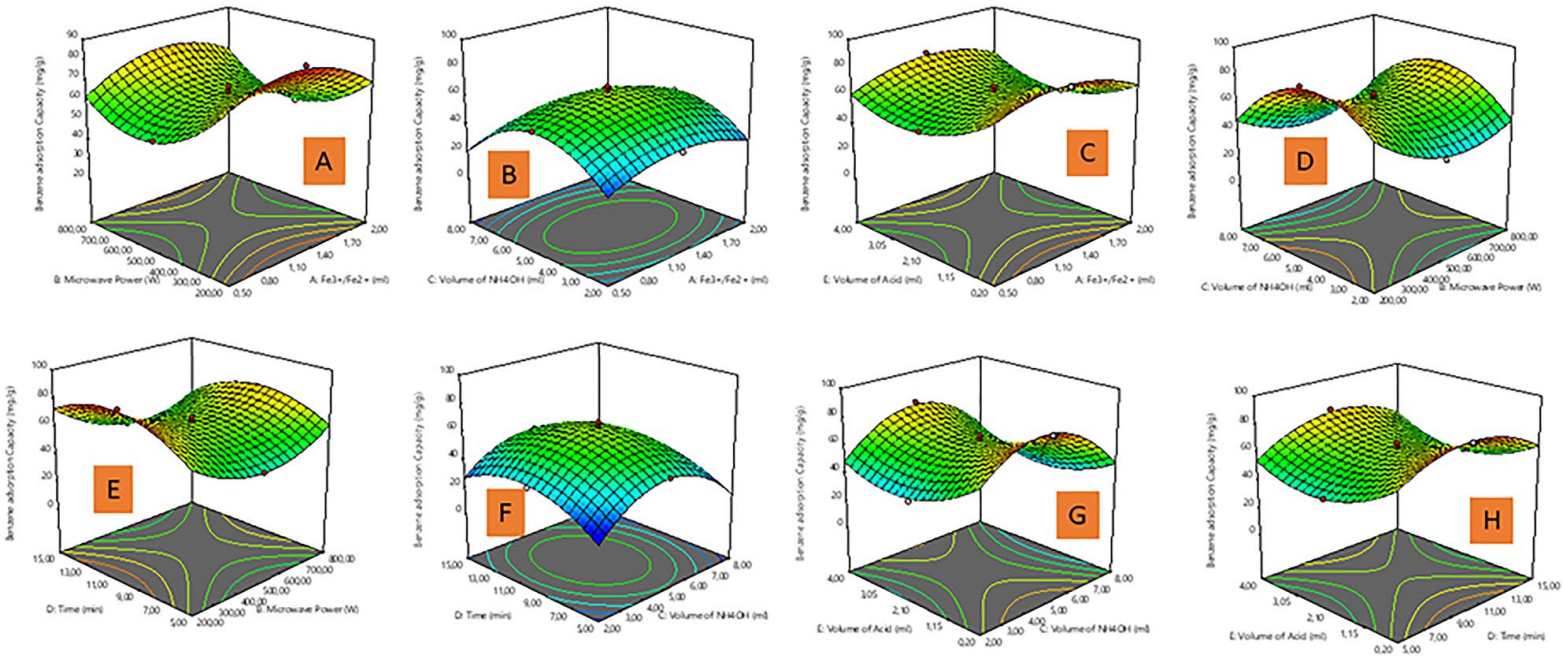
3D response plots of (**A**) Fe^3+/^Fe^2+^ solution and microwave power, (**B**) Fe^3+/^Fe^2+^ solution and volume of ammonium hydroxide, (**C**) Fe^3+/^Fe^2+^ solution and volume of oleic acid, (**D**) microwave power and volume of ammonium hydroxide, (**E**) microwave power and time, (**F**) volume of ammonium hydroxide and time, (**G**) volume of ammonium hydroxide and volume of oleic acid, (**H**) time and volume of oleic acid on benzene adsorption capacity (BAC).

The three-dimensional (3D) surface plot was used to understand the interactive relationships between preparation variables, and the responses of the model^37,38^. In Fig. 2, a-3D response surface plots depict the significant interaction between the preparation parameters (Fe^3+^/Fe^2+^ solution, microwave power (MP), reaction time, and volume of oleic acid (VOA)) on % Fe content in accordance with the ANOVA (Table S1) are discussed. In Fig. 2A, the % Fe content of the MNP-OA composite increased with an increase in Fe^3+^/Fe^2+^ solution against the time from 0.5 to 1.25 ml, after 1.5 mL the % Fe content became constant. Though an increase or decrease in Fe^3+^/Fe^2+^ solution versus VOA has no significant influence on the % Fe content (Fig. 2B). In Fig. 2C,D,E, the significant effect of microwave power (MP) against other parameters (volume of ammonia water (VAW), reaction time, and VOA) on % Fe content is evident, which aligns with the high F value in ANOVA (Table S1). The % Fe content of the MNP-OA composite decreased with an increment in MP from 200 to 600 W, then steadily increased from 600 to 800 W. The highest % Fe content was obtained at 200 W. The % Fe content reduction with an increase in MP, is attributed to the microwave irradiation power augmenting the reaction temperature, thus generates dielectric heating which raises the temperature of the solution, resulting in MNP-OA with large particle size^39^. Herein, the microwave energy is transferred selectively to the microwave absorbing material (MNP-OA composite) in the form of heat energy, thus the intrinsic temperature around the ion is much higher, resulting in the increase in collision, and formation of aggregated magnetite nuclei^40^. Such localized heating of ions controls the nucleation rate; therefore the magnetite nanoparticles control the localized heating rate of ions, and results to the increased crystallite size due to increased magnetite nanoparticles^39,41^. Figure 2B,E,G,H, shows that the % Fe content of the MNP-OA composite increased with an increase in the VOA from 0.2 to 2.10 mL, the % Fe content decreased as VOA volume reached 2.10 mL. The optimum VOA was observed at 0.2 mL. The VOA plays a strong role in the % Fe content in line with the high F values as presented in Table S1, the high VOA may interfere with particle growth, and distribution^42,43^. Figure 2A,D,F,H, shows that increasing the reaction time from 5 to 10 min increased the % Fe content of the MNP-OA composite to a certain point then slightly decreased at a high reaction time (15 min). The optimum time for high % Fe content was 10 min. As time rarely affect the reaction process, in comparison to the VOA, Fe^3+^/Fe^2+^ solution, and MP with a high F values from the ANOVA (Table S1). Also, an increase or decrease in VAW against other parameters (reaction time, MP, and VOA) has no considerable influence on the % Fe content of the MNP-OA composite as presented in Fig. 2C,F,G. This further justifies insignificant influence of VAW as evidenced in ANOVA (Table S1). The VOA plays an enormous influential role in the high % Fe content, followed by Fe^3+^/Fe^2+^ solution, MP and reaction time, which is in accordance with the high F values.

In Fig. 3, a-3D response surface plots depict the significant interaction between the preparation parameters (microwave power (MP), Volume of ammonia water (VAW), reaction time, and volume of oleic acid (VOA)) on benzene adsorption capacity (BAC) in accordance with the ANOVA in Table S2. The MP significantly influences BAC (high F value from the ANOVA in Table S2). The BAC of the MNP-OA composite increased with an increment in MP (Fig. 3A,D,E) from 200 to 500 W amongst other parameters (Fe^3+^/Fe^2+^ solution, VAW, and time). The highest BAC for MNP-OA composite is at 200 W, whilst further increase in MP results in the reduction of BAC. At higher MP, the MNP-OA composite particle size becomes bigger due to high viscosity, and nucleation. In general, the nucleation, and subsequent growth of nanoparticles is very fast, and thus results in larger shaped particles, which can be related to high intensity energy in the microwave oven^44^. Figure 3B,D,F,G, shows that the BAC of the MNP-OA composite increased with an increment in the VAW from 2 to 8 mL, as the optimum volume is at 5 mL and beyond this volume the BAC begins to decrease. This further signify that low and high VAW amount are not good for the BAC. Since the VAW amount influences the degree of oxidation in the formation of MNP-OA composite, hence lower VAW results in mixed phase of Fe_2_O_3_, whilst higher VAW leads to pure form of Fe_3_O_4_ with large particle size^43^. The VAW was the most significant parameter for BAC due to its large F-value in the ANOVA (Table S2). Moreover, increasing the reaction time against other parameters (VOA, VAW, and MP) from 5 to 10 min as presented in Fig. 3E,F,H, increased the BAC of the MNP-OA composite to a certain point then slightly decreased at a high reaction time of 15 min, whilst the optimum time is 5 min. Additionally, the BAC was also observed to increase in Fig. 3C,G,H with an increase in the VOA from 0.2 to 2.10 mL, as the optimum volume is at 0.2 mL and beyond this point the BAC begins to decrease. Herein, the VOA amount affects the dispersibility and size of MNP-OA composite, which directly affects the BAC in this study^45^. The other parameters (VAW and reaction time) against VOA in Fig. 3G,H significantly influences the increase or decrease of BAC, as compared with negligible influence of Fe^3+/^Fe^2+^ solution (due to its insignificancy as presented in Table S2) against VAW, and VOA as presented in Fig. 3B,C.

**Figure 4:**
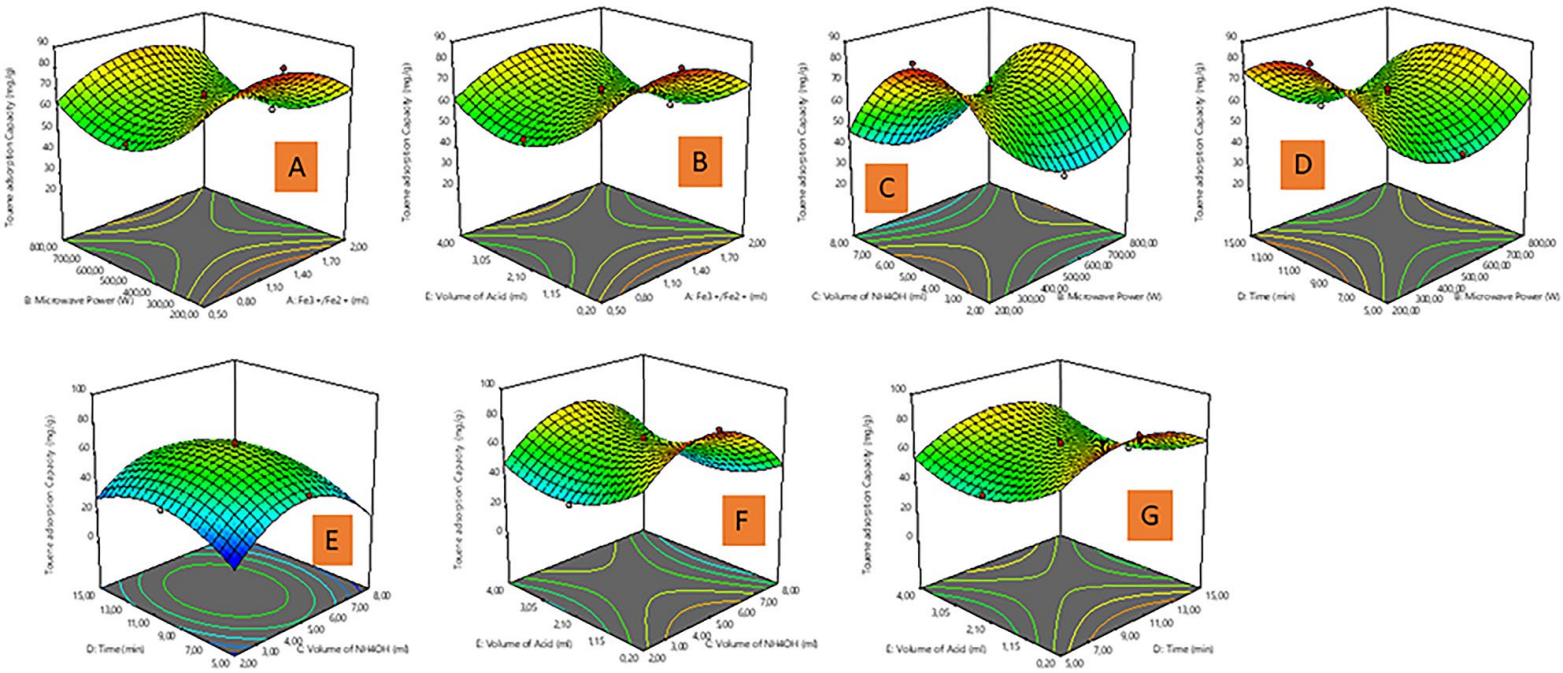
3D response plots of (**A**) Fe^3+/^Fe^2+^ solution and microwave power, (**B**) Fe^3+/^Fe^2+^ solution and volume of oleic acid, (**C**) microwave power and volume of ammonium hydroxide, (**D**) microwave power and time, (**E**) volume of ammonium hydroxide and time, (**F**) volume of ammonium hydroxide and volume of oleic acid, (**G**) time and volume of oleic acid on toluene adsorption capacity (TAC).

Figure 4A,C,D shows toluene adsorption capacity (TAC) changes between MP and other parameters (Fe^3+/^Fe^2+^ solution, VAW and reaction time). As presented in Fig. 4A,C,D, increasing the MP values, resulted in TAC increment, and it has significant impact based on high F value as presented in Table S3. Meanwhile, other parameters mentioned above shows significant influence on TAC as well, but the VAW exhibited more impact on TAC amongst other parameters (Fig. 4C,E,F) in this study. Herein, by increasing the VAW from 2 to 8 mL, the TAC also increases, whilst the optimum volume is at 5 mL and beyond this volume the TAC begins to decrease. The VAW has a direct influence on TAC in this study, as well as other variables (MP, reaction time, and VOA) interacting with it, also thus influence TAC as presented in Fig. 4C,E,F. The effect of time on TAC is also highlighted as presented in Fig. 4D,E,G, whereby as the reaction time is increased against other parameters (MP, VAW, and V0A) from 5 to 10 min, the TAC of the MNP-OA composite also rises to a certain point then slightly decreased at a high reaction time. However, both the MP, VAW, and VOA as parameters in this scenario has direct influence on enhanced TAC by MNP-OA composite as presented in Fig. 4D,E,G. In Fig. 4B,F,G, the interaction of VOA against Fe^3+/^Fe^2+^ solution, VAW, and reaction time on TAC were also studied. The results show that VOA increment from 0.2 to 2.10 mL, results in an enhanced TAC which is a direct increase. Furthermore, the significant effects of VOA in Fig. 4B,F and Time (Fig. 4G) on TAC from the 3D surface plots is more than the influence of Fe^3+/^Fe^2+^ solution in this study.

**Figure 5:**
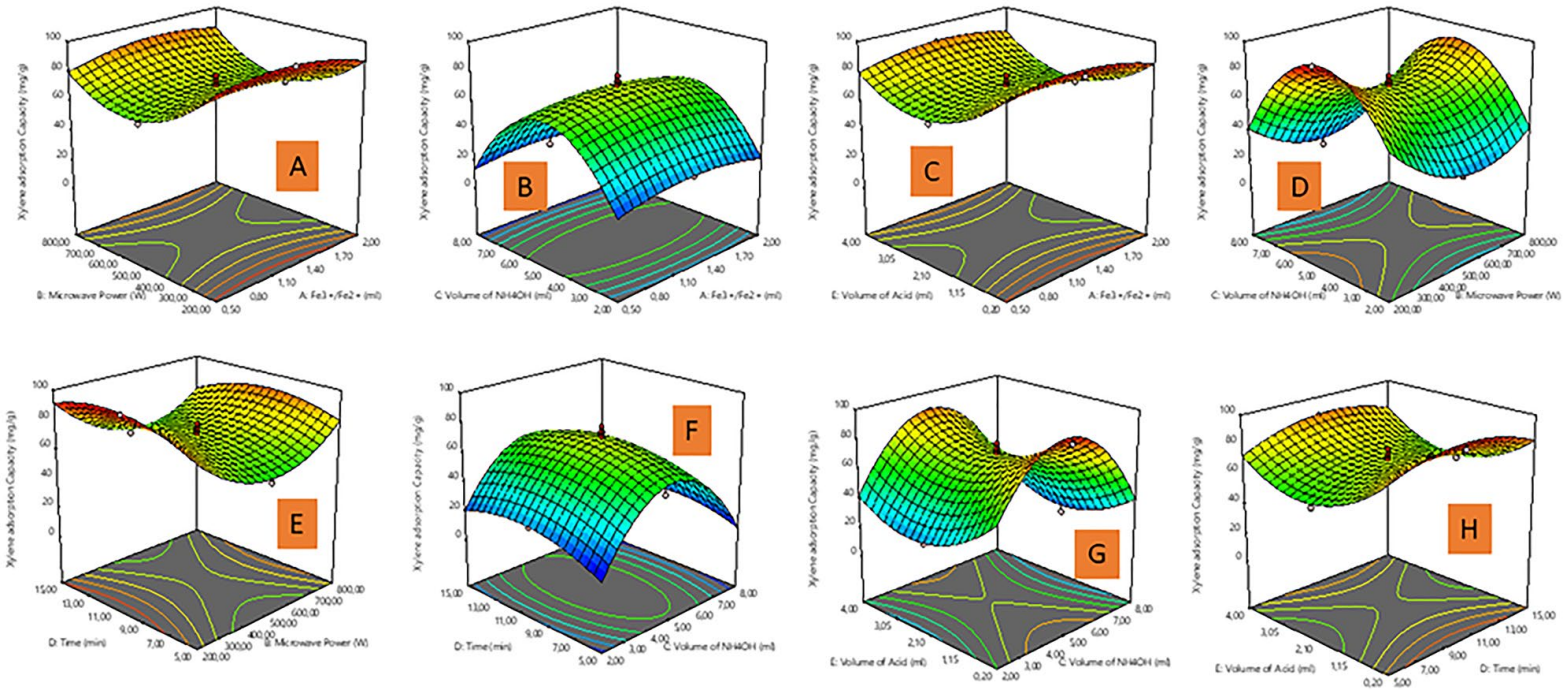
3D response plots of (**A**) Fe^3+/^Fe^2+^ solution and microwave power, (**B**) Fe^3+/^Fe^2+^ solution and volume of ammonium hydroxide, (**C**) Fe^3+/^Fe^2+^ solution and volume of oleic acid, (**D**) microwave power and volume of ammonium hydroxide, (**E**) microwave power and time, (**E**) volume of ammonium hydroxide and time, (**F**) volume of ammonium hydroxide and time, (**G**) volume of ammonium hydroxide and volume of oleic acid, (**H**) time and volume of oleic acid on xylene adsorption capacity (XAC).

Figure 5A,D,E shows the interaction of MP against variables such as Fe^3+/^Fe^2+^ solution, VAW, reaction time on XAC by MNP-OA composite. The results showed that MP increased the XAC, which is similar to benzene adsorption capacity (BAC) analysis as presented in Fig. 3A,D,E. However, both VAW and reaction time also influence on the XAC in Fig. 5D,E, in comparison to insignificant impact of Fe^3+^/Fe^2+^ solution (Fig. 5A) on XAC. Figure 5B,D,F,G, shows that the XAC of the MNP-OA composite increased with an increment in the VAW from 2 to 8 mL, against other parameters (Fe^3+/^Fe^2+^ solution, MP, time, and VOA). Moreover, similar results obtained for XAC, were noticed for BAC as presented in Fig. 3B,D,F,G. The XAC of the MNP-OA composite changes with an increase in the reaction time against other parameters (MP, VAW, and VOA) from 5 to 10 min to a certain point then slightly decreased at a high reaction time as presented in Fig. 5E,F,H. In addition, Fig. 5C,G,H presented that the XAC of MNP-OA composite increase with an increment in the VOA from 0.2 to 2.10 mL, as the optimum volume is at 0.2 mL and beyond this point the XAC begins to decrease. Similar observation with reaction time, and VOA influence on XAC, were identical with BAC in Fig. 3E,F,H and Fig. 3C,G,H, respectively. Overall, the VAW, followed by VOA, and MP are significant factor that influences BAC, TAC and XAC respectively, which aligns with the ANOVA datas as presented in Table S2, S3, S4, and also has not been discussed so far from any previous literature. 3.3 Optimization of preparation variables on % Fe content and BAC, TAC and XAC.

From the 3D response plots interaction of independent variables on % Fe content, the BAC, TAC and XAC, the most significant parameters for the responses and specific levels were determined. The ideal conditions for Fe^3+/^Fe^2+^ solution, VAW, MP, VOA and time were utilized without any hindrance, as presented in Table 3 with the optimum parameters, and respective maximum values for % Fe content, BAC, TAC and XAC respectively. Based on optimized conditions, the solution with the highest desirability from the numerical optimization was taken into consideration. As presented in Table 3, the observed (experimental)values are in good agreement with the predicted values, while the deviation difference were in the range of 0.28 – 1.77%. The optimum values for maximum % Fe content, BAC, TAC and XAC were found to be: 85.57%, 90.02 mg/g, 90.07 mg/g, and 96.31 mg/g respectively.

**Table 3:** Optimization constraints for MNP-OA composite, % Fe content and its performance on Benzene, toluene and xylene adsorption capacity.

Parameter conditions							Responses	
Responses	Quality specification (QS)	A: Fe^3+/^Fe^2+^ solution	B: Microwave Power	C: Volume of ammonia water	D: Time	E: Volume of Acid	Observed (experimental)	Predicted
% Fe Content	QS 1	1.52	200	5.43	10.51	0.20	85.62	85.15
	QS 2	1.93	200	5.20	9.11	0.20	85.52	85.23
BAC	QS 1	1.93	200	5.24	10.21	0.20	89.49	87.92
	QS 2	1.80	200	5.77	10.26	0.20	90.55	88.92
TAC	QS 1	1.96	200	4.94	10.27	0.20	89.93	89.58
	QS 2	1.97	200	4.69	9.71	0.20	90.22	89.61
XAC	QS 1	0.50	200	2.46	9.02	0.20	97.26	97.25
	QS 2	0.50	200	2.46	9.04	0.20	95.35	94.83

### Characterization

#### XRD

**Figure 6:**
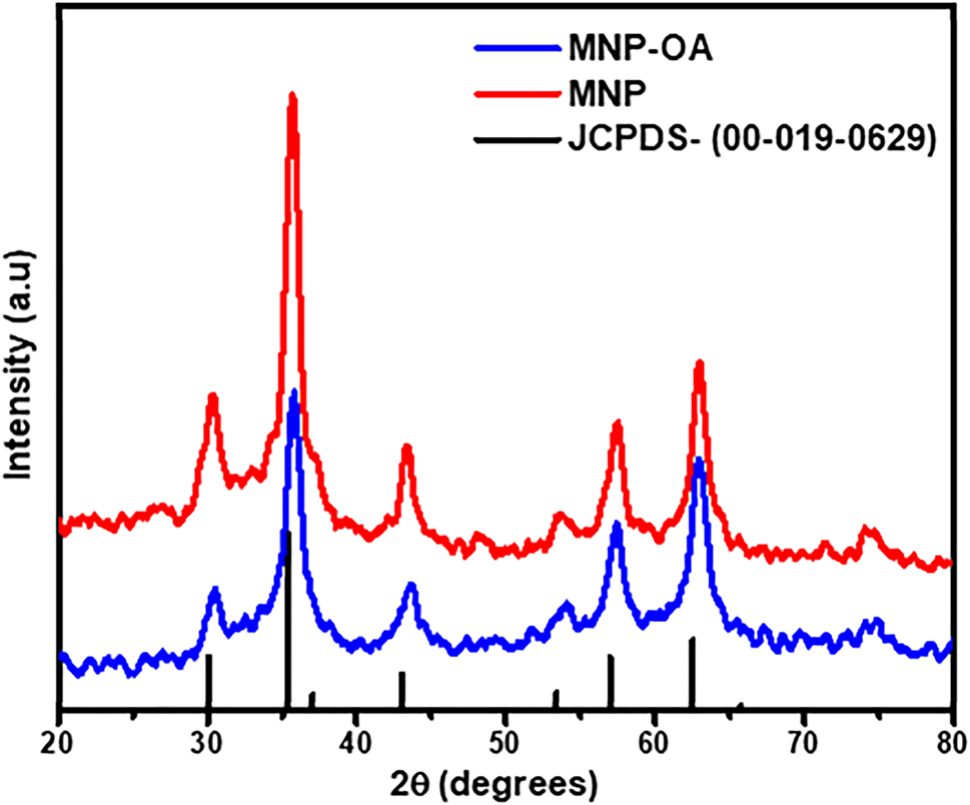
XRD pattern of magnetite (MNP) and magnetite-oleic acid (MNP-OA) composite.

**Figure 7:**
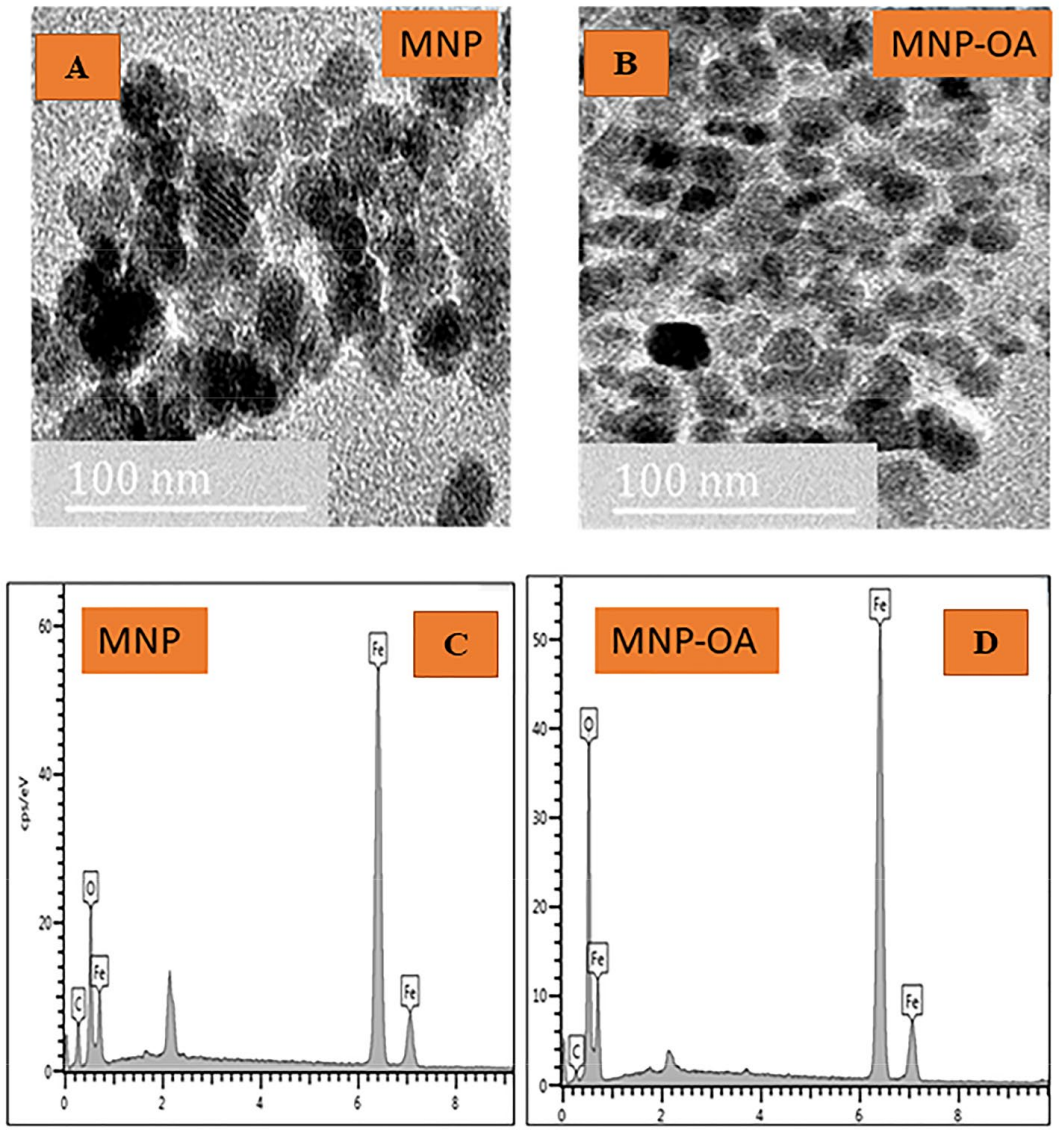
TEM images and EDX of pure MNP (**A**,**C**), and MNP-OA (**B**,**D**) composite.

The XRD patterns of MNP-OA sample, and pure MNP, are compared to the standard pattern of MNP and the spectra are presented in Fig. 6. The intensities of all the peaks in the XRD patterns of the two samples match very well with standard Fe_3_O_4_ powder diffraction card (19-0629)^46^ indicating that the samples had crystalline structure without any impurities. The crystallite sizes of the nanoparticles were calculated using the Scherrer equation from the most intense (311) peak. The average crystallite size of the MNP, and MNP-OA samples are 19.7 and 17.1 nm respectively. This observation clearly evidences the significance of VOA factor based on RSM modelling, in uniformity of MNP-OA in terms of very narrow size distribution, and low nanometre range sizes, which are basic requirements for enhanced adsorptive removal of pollutants^47^. The results also conforms in line with TEM results as further discussed below in Fig. 7.

#### TEM and EDX analysis

The transmission electron spectroscopy (TEM) of the MNP and MNP-OA composite are presented in Fig. 7A,B. Both the MNP and MNP-OA nanoparticles had a spherical shape however, a strong magnetic dipole–dipole interaction was shown by uncoated magnetite causing big clusters and bigger particle size^19^. The agglomeration in the nanometric size particles of the MNP was due to the absence of surface capping agent^45,48^. After surface modification with the oleic acid (Fig. 7B), the nanoparticles were better separated from one another as compared with the MNP (Fig. 7A), due to the presence of the carboxylic acid surfactant on the surface of the nanoparticles. A non-magnetic layer was formed on the magnetite surface resulting in a decrease in the particle size of the MNP-OA material. The particles in the MNP-OA had less degree of aggregation, and presented a better isolation due to the strongly held uniform monolayer coverage of oleic acid^48^. The reduced agglomeration will significantly increase the BTX removal capacity, as presented in “Adsorption dynamics studies, S2”, and thus increases the scope of MNP-OA material application for further adsorption studies. The average particle size of the MNP and MNP-OA composite were 18.4 ± 0.5 nm and 15.6 ± 0.5 nm respectively. These results are comparable with the XRD results (Fig. 6) which show nanosized material.

The elemental analysis results of the MNP and MNP-OA samples obtained from EDX are shown in Fig. 7C,D. The EDX analysis confirms that the main constituent elements of pure MNP and MNP-OA to be iron (Fe), carbon (C), and oxygen (O)^48^. Figure 7C,D shows peaks around 0.8, 6.3, and 6.8 keV associated with the binding energies of Fe^49^. The spectra of pure MNP and MNP-OA composite both contained two major peaks, which were assigned to Fe and O and further affirm synthesis of these materials. The spectrum of the pure MNP with small peak C is attributed to the ethanol used during synthesis and ethanol used for washing the nanoparticles. The MNP-OA composite contained three major peaks assigned not only for Fe and O but also for C. The carbon peak observed for the magnetite-organic acid composite confirmed the oleic acid layer on the surface of the magnetite^50^. As shown in Fig. 7C,D, and also Table 4, the EDX graphs for the samples show that the % Fe content for MNP-OA composite were lower than that of pure MNP. This result can be attributed to the presence of the non-magnetic layer immobilized on the surface of the pure magnetite nanoparticles^42^.

**Table 4:** Elemental analysis of pure magnetite (MNP) and oleic coated magnetite composite (MNP-OA).

Sample	Fe	O	C
MNP	78.8	16.35	4.85
MNP-OA	68.42	27.08	4.50

**Figure 8:**
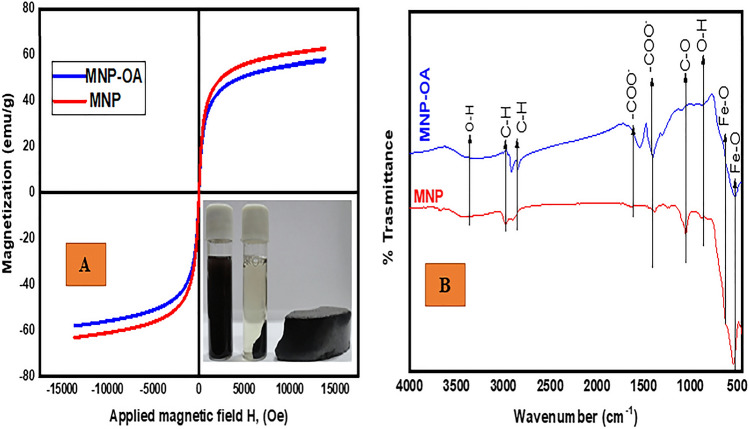
(**A**) VSM curve, and (**B**) FTIR spectra of MNP, and MNP-OA composite.

Figure 8A shows the magnetization curves of the synthesized pure MNP (a) and MNP-OA (b) composite. MNP had a saturation magnetization (M_S_) value of 62.9 emu/g which is lower than that of the bulk MNP (92 emu/g). The decrease in magnetization of the synthesized MNP compared to that of bulk MNP was attributed to the small particle size of the nanoparticles^33,51^. For the MNP-OA composite, magnetization was achieved at 59 emu/g, with lower M_S_ value compared to that of bare MNP The decrease of saturation magnetization MNP-OA compared to the pure MNP was due to the presence of the non-magnetic OA surface coating^52^. In addition, coating the MNP with OA limits the former from the oxidation therefore decreasing the magnetic dipole–dipole interaction between the particles^52,53^. The illustration presented in Fig. 8A indicates that MNP, and MNP-OA materials were easily separated by an external magnetic field.

The study further investigated the FTIR spectra of MNP, and MNP-OA, an interaction between the functional groups of the OA and MNP were confirmed, as presented in Fig. 8B. The MNP spectrum in Fig. 8B, depicts that the Fe–O band was confirmed by a shoulder at 624 cm^−1^ and a peak at 544 cm^−1^^54,55^. The hydroxyl group (O–H) due to water on the nanoparticles was achieved at 3429 cm^−1^^48,55^. The strong adsorption at 1052 cm^−1^ was due to C–O single bond stretching. The spectrum further presents other peaks at around 870 and 984 cm^−1^ which were a result of the two solvents used (deionised water and ethanol) during the study^56^. However, it was observed that after modification of the MNP with OA, there was shifting, disappearance and reduction in intensities of some of the characteristic peaks. For instance, the reduction of the peak at 3429 cm^−1^ was attributed to the displacement of water by the oleic acid. Likewise, the shift of the asymmetric and symmetric CH stretching of the OA coating agent to much lower frequency region further indicated that the hydrocarbon chains in the monolayer surrounding the nanoparticles were in a closed-packed, crystalline state^49^. It was also observed that coating the long-chain carboxylic acid (OA) into the MNP resulted in a hydrophobic composite (MNP-OA) material^48^. Other peaks that show a slight shift were the Fe–O occurring at 624 and 544 cm^−1^_._ The shifting of Fe–O stretching frequency to a lower region was a result of the interaction between the Fe atoms (MNP) and the carboxyl groups from the OA which causes partial single bond character of the carbonyl C=O group^57,58^_._ Lastly the shift which occurred at 1407 to 1404 cm^−1^ and 1539 to 1534 cm^−1^, which are ascribed to asymmetric and symmetric carboxylic ion (COO^−^) indicate the strong interaction between MNP and OA^59^. Clearly, the significant influence of VOA amongst other variables from the RSM model, impacted more functional groups on MNP-OA composite thus providing new active sites, and more functional groups for enhanced BTX adsorption in this study, as further presented in S2.

## Conclusion

The MNP-OA composite preparation parameters (molar volume of Fe^3+^/Fe^2+^ solution, MP, VAW, reaction time, and VOA) through MAS approach were successfully optimized using RSM design, on the maximum % Fe content, BAC, TAC and XAC respectively. In accordance with the statistical analysis, the VOA had a more significant effect on % Fe content, followed by molar volume of Fe^3+^/Fe^2+^ solution, MP, and reaction time. The VAW had more influence on BAC, TAC, and XAC in this study based on high F values from the ANOVA in comparison to VOA, MP, and time. These significant synthesis variables also influence the physicochemical properties of MNP-OA composite judging from the XRD and TEM analysis in this study. The optimum experimental results for % Fe content, BAC, TAC and XAC were 85.57%, 90.02 mg/g, 90.07 mg/g, and 96.04 mg/g respectively, as there was no considerable difference in obtained experimental data and the predicted data. The present experimental results suggested that process optimization by RSM is an efficient approach to obtain maximum % Fe content, enhanced BAC, TAC, XAC removal through microwave assisted synthesis. BTX adsorption dynamic studies revealed high capacity at solution pH of 7 for MNP and solution pH 8 MNP-OA and 0.1 g adsorbent dose.

## Supplementary Information

Supplementary Information.

## Data Availability

The data used/presented in this current study are openly available from the corresponding author on reasonable request.

## References

[1] Mohammed J, Nasri NS, Ahmad Zaini MA, Hamza UD, Ani FN (2015). Adsorption of benzene and toluene onto KOH activated coconut shell based carbon treated with NH_3_. Int. Biodeterior. Biodegradation.

[2] Anjum H (2019). A review on adsorptive removal of oil pollutants (BTEX) from wastewater using carbon nanotubes. J. Mol. Liq..

[3] Bayode AA (2021). Tuning ZnO/GO pn heterostructure with carbon interlayer supported on clay for visible-light catalysis: Removal of steroid estrogens from water. Chem. Eng. J..

[4] Farsouni Eydi E, Shariati A, Khosravi-Nikou MR (2019). Separation of BTEX compounds (benzene, toluene, ethylbenzene and xylenes) from aqueous solutions using adsorption process. J. Dispers. Sci. Technol..

[5] Saha D, Mirando N, Levchenko A (2018). Liquid and vapor phase adsorption of BTX in lignin derived activated carbon: Equilibrium and kinetics study. J. Clean. Prod..

[6] Mèçabih Z (2017). Adsorption-desorption of BTX (benzene, toluene and O-xylene) on Fe, Fe-Al pillared clay. J. Encapsulation Adsorp. Sci..

[7] Lu F, Astruc D (2018). Nanomaterials for removal of toxic elements from water. Coord. Chem. Rev..

[8] Kostyukhin EM (2020). Microwave-assisted synthesis of water-dispersible humate-coated magnetite nanoparticles: Relation of coating process parameters to the properties of nanoparticles. Nanomater..

[9] Ofomaja AE, Naidoo EB, Pholosi A (2020). Intraparticle diffusion of Cr (VI) through biomass and magnetite coated biomass: A comparative kinetic and diffusion study. South Afri. J. Chem. Eng..

[10] Abbas SH, Ismail IM, Mostafa TM, Sulaymon AH (2014). Biosorption of heavy metals: A review. J. Chem. Sci. Technol..

[11] Al-Hussain SA, Ezzat AO, Gaffer AK, Atta AM (2018). Removal of organic water pollutant using magnetite nanomaterials embedded with ionic copolymers of 2-acrylamido-2-methylpropane sodium sulfonate cryogels. Polym. Inter..

[12] Fleck L (2018). Efficiency of the electrochemical treatment of textile effluent using two configurations of sacrificial electrodes. Holos Environ..

[13] Natarajan S (2019). Multifunctional magnetic iron oxide nanoparticles: Diverse synthetic approaches, surface modifications, cytotoxicity towards biomedical and industrial applications. BMC Mater..

[14] Ouma IL, Naidoo EB, Ofomaja AE (2017). Iron oxide nanoparticles stabilized by lignocellulosic waste as green adsorbent for Cr (VI) removal from wastewater. EPJ Appl. Phys.

[15] Karimi Z, Karimi L, Shokrollahi H (2013). Nano-magnetic particles used in biomedicine: Core and coating materials. Mater. Sci. Eng: C.

[16] Araújo-Neto RP (2014). Monodisperse sodium oleate coated magnetite high susceptibility nanoparticles for hyperthermia applications. J. Magn. Magn. Mater..

[17] Liu J, Dai C, Hu Y (2018). Aqueous aggregation behavior of citric acid coated magnetite nanoparticles: Effects of pH, cations, anions, and humic acid. Environ. Res..

[18] Pholosi A, Naidoo BE, Ofomaja AE (2018). Clean application of magnetic biomaterial for the removal of As (III) from water. Environ. Sci. Pollut. Res..

[19] Shete PB, Patil RM, Tiwale BM, Pawar SH (2015). Water dispersible oleic acid-coated Fe_3_O_4_ nanoparticles for biomedical applications. J. Magn. Magn. Mater..

[20] Masuku M, Ouma L, Pholosi A (2021). Microwave assisted synthesis of oleic acid modified magnetite nanoparticles for benzene adsorption. Environ. Nanotechnol. Monit. Manag..

[21] Pourgolmohammad B, Masoudpanah SM, Aboutalebi MR (2017). Synthesis of CoFe_2_O_4_ powders with high surface area by solution combustion method: Effect of fuel content and cobalt precursor. Ceram. Int..

[22] Moosavi S (2017). Hydrothermal synthesis, magnetic properties and characterization of CoFe_2_O_4_ nanocrystals. Ceram. Int..

[23] Kalyani S, Sangeetha J, Philip J (2016). Effect of precipitating agent and solvent polarity on the size and magnetic properties of magnetite nanoparticles prepared by microwave assisted synthesis. J. Nanosci. Nanotechnol..

[24] Zhu Y-J, Chen F (2014). Microwave-assisted preparation of inorganic nanostructures in liquid phase. Chem. Rev..

[25] Ali A (2016). Synthesis, characterization, applications, and challenges of iron oxide nanoparticles. Nanotechnol. Sci. Appl..

[26] Gharibshahian M, Mirzaee O, Nourbakhsh M (2017). Evaluation of superparamagnetic and biocompatible properties of mesoporous silica coated cobalt ferrite nanoparticles synthesized via microwave modified Pechini method. J. Magn. Magn. Mater..

[27] Fariñas J (2018). Microwave-assisted solution synthesis, microwave sintering and magnetic properties of cobalt ferrite. J. Eur. Ceram. Soc.

[28] Sanni S, Viljoen E, Ofomaja A (2019). Accelerated electron transport and improved photocatalytic activity of Ag/AgBr under visible light irradiation based on conductive carbon derived biomass. Catal. Lett..

[29] Ramos Guivar JA (2018). Adsorption of arsenite and arsenate on binary and ternary magnetic nanocomposites with high iron oxide content. Appl. Surf. Sci..

[30] Ryu C (2022). Highly optimized iron oxide embedded poly (lactic acid) nanocomposites for effective magnetic hyperthermia and biosecurity. Int. J. Nanomedicine.

[31] Mosafer J, Abnous K, Tafaghodi M, Jafarzadeh H, Ramezani M (2017). Preparation and characterization of uniform-sized PLGA nanospheres encapsulated with oleic acid-coated magnetic-Fe_3_O_4_ nanoparticles for simultaneous diagnostic and therapeutic applications. Colloids Surf. A: Physicochem. Eng. Asp..

[32] Mahdavi M (2013). Synthesis, surface modification and characterisation of biocompatible magnetic iron oxide nanoparticles for biomedical applications. Molec..

[33] Ouma L, Ofomaja AJRA (2020). Probing the interaction effects of metal ions in Mn_x_Fe_(3__−__x)_O_4_ on arsenite oxidation and adsorption. RSC Adv.

[34] Sanni S, Viljoen E, Ofomaja A (2020). Design of ordered Ag/AgBr nanostructures coupled activated carbon with enhanced charge carriers separation efficiency for photodegradation of tetracycline under visible light. J. Mol. Liq..

[35] Sanni S, Viljoen E, Ofomaja A (2021). Tailored synthesis of Ag/AgBr nanostructures coupled activated carbon with intimate interface interaction for enhanced photodegradation of tetracycline. Process Saf. Environ. Prot.

[36] Ghorbannezhad P, Firouzabadi MD, Ghasemian A, de Wild PJ, Heeres H (2018). Sugarcane bagasse ex-situ catalytic fast pyrolysis for the production of Benzene, Toluene and Xylenes (BTX). J. Anal. Appl. Pyrolysis.

[37] Ani J (2019). Application of response surface methodology for optimization of dissolved solids adsorption by activated coal. Appl. Water Sci..

[38] Sanni SO, Viljoen EL, Ofomaja AE (2021). Tailored synthesis of Ag/AgBr nanostructures coupled activated carbon with intimate interface interaction for enhanced photodegradation of tetracycline. Process Saf. Environ. Protec..

[39] Riaz S, Ashraf R, Akbar A, Naseem S (2014). Microwave assisted iron oxide nanoparticles—Structural and magnetic properties. IEEE Trans. Magn..

[40] Al-Gaashani R, Radiman S, Tabet N, Daud AR (2011). Effect of microwave power on the morphology and optical property of zinc oxide nano-structures prepared via a microwave-assisted aqueous solution method. Mater. Chem. Phys..

[41] Acharya S, Singh K (2011). Microwave-assisted chemical reduction routes for direct synthesis of Fe–Pt nanoparticles in ordered face centered tetragonal L1 0 phase. Appl. Nanosci..

[42] Zuluaga-Parra JD (2020). A novel method for the modification of magnetite nanoparticles for the enhancement of its dispersibility in hydrophobic media. J. Magn. Magn. Mater..

[43] Zheng Y-Y (2020). Controllable synthesis of monodispersed iron oxide nanoparticles by an oxidation-precipitation combined with solvothermal process. Mater. Chem. Phys..

[44] Torres-Gómez N (2019). Shape tuning of magnetite nanoparticles obtained by hydrothermal synthesis: Effect of temperature. J. Nanomater..

[45] Neto WS (2018). Superparamagnetic nanoparticles stabilized with free-radical polymerizable oleic acid-based coating. J. Alloys Compd.

[46] Pholosi A, Naidoo EB, Ofomaja AE (2019). Enhanced arsenic (III) adsorption from aqueous solution by magnetic pine cone biomass. Mater. Chem. Phys.

[47] Okoli CP, Ofomaja AE (2019). Development of sustainable magnetic polyurethane polymer nanocomposite for abatement of tetracycline antibiotics aqueous pollution: Response surface methodology and adsorption dynamics. J. Clean. Prod.

[48] Muthukumaran T, Philip J (2016). Effect of phosphate and oleic acid capping on structure, magnetic properties and thermal stability of iron oxide nanoparticles. J. Alloys Compd.

[49] Mahdavi M (2013). Synthesis, surface modification and characterisation of biocompatible magnetic iron oxide nanoparticles for biomedical applications. Molecules.

[50] Velusamy P (2016). Synthesis of oleic acid coated iron oxide nanoparticles and its role in anti-biofilm activity against clinical isolates of bacterial pathogens. J. Taiwan Inst. Chem. Eng..

[51] Toyos-Rodríguez C (2019). A simple and reliable synthesis of superparamagnetic magnetite nanoparticles by thermal decomposition of Fe(acac)_3_. J. Nanomater.

[52] Viante MF, Zanela TMP, Stoski A, Muniz EC, Almeida CAP (2018). Magnetic microspheres composite from poly (ethylene terephthalate) (PET) waste: Synthesis and characterization. J. Clean. Prod.

[53] Anbarasu M, Anandan M, Chinnasamy E, Gopinath V, Balamurugan K (2015). Synthesis and characterization of polyethylene glycol (PEG) coated Fe_3_O_4_ nanoparticles by chemical co-precipitation method for biomedical applications. Spectrochim. Acta A Mol Biomol. Spectro..

[54] Iyengar SJ, Joy M, Maity T, Chakraborty J, Kotnala RK, Ghosh S (2016). Colloidal properties of water dispersible magnetite nanoparticles by photon correlation spectroscopy. RSC Adv..

[55] Soares PIP (2014). Effects of surfactants on the magnetic properties of iron oxide colloids. J. Colloid Interface Sci.

[56] Chaki SH, Malek TJ, Chaudhary MD, Tailor JP, Deshpande MP (2015). Magnetite Fe_3_O_4_ nanoparticles synthesis by wet chemical reduction and their characterization. Adv. Nat. Sci. Nanosci. Nanotechnol..

[57] Yang K, Peng H, Wen Y, Li N (2010). Re-examination of characteristic FTIR spectrum of secondary layer in bilayer oleic acid-coated Fe_3_O_4_ nanoparticles. Appl. Surf. Sci.

[58] Li D (2010). An easy fabrication of monodisperse oleic acid-coated Fe_3_O_4_ nanoparticles. Mater. Lett.

[59] Petcharoen K, Sirivat A (2012). Synthesis and characterization of magnetite nanoparticles via the chemical co-precipitation method. Mater. Sci. Eng. C: B.

