# Supplementary material for: Optimization studies of BTX removal by magnetite coated oleic acid obtained from microwave-assisted synthesis using response surface methodology
Source: Sci Rep. 2022 Nov 3;12:18609. doi: 10.1038/s41598-022-22716-w (PMC9633638; doi:10.1038/s41598-022-22716-w)
Supplement: Supplementary file 1 — Supplementary Information. [file 41598_2022_22716_MOESM1_ESM.docx]

**Optimization Studies of BTX Removal by Magnetite Coated Oleic Acid produced from Microwave-** **Assisted Synthesis using Response Surface Methodology**

**Makhosazana Masuku, Linda Ouma, Saheed Sanni^*^, Agnes Pholosi^*^,**

Biosorption and Water Treatment Research Laboratory, Vaal University of Technology, Private Bag X021, Vanderbijlpark 1900, South Africa

* Corresponding author: [agnesp@vut.ac.za](mailto:agnesp@vut.ac.za); mosqit.saheed@gmail.com

**S1: Batch adsorption studies on removal of BTX**

Benzene, toluene, and xylene (BTX) batch adsorption tests were carried out utilizing the synthesized MNP-OA nanocomposites. The MNP-OA nanocomposites (0.1 g) were added unto 250 mL container flask comprising of 50 mL, 100 mg/L benzene, toluene, and xylene solutions respectively. The solutions were further sonicated for one hr prior, the transfer inside a “Tornado machine” for adsorption experiment, at agitation speed of 300 rpm, amidst room temperature conditions for 2 h. After 2 h agitation, the solutions were then centrifuged, and separated using 0.45 µm filters.

The concentration of BTX species in solution was analysed using a Perkin-Elmer (USA) Lambda 25 UV-visible spectrometer to determine the amounts of benzene, toluene, and xylene left in the solution, measured at their maximum wavelength of 253, 260 and 267 nm respectively. The adsorption capacity for BTX solution adsorbed per gram of MNP-OA nanocomposites at equilibrium was calculated according to the expression below:

$\boldsymbol{Q}_{\boldsymbol{e}}$**=** $\frac{\boldsymbol{(}\boldsymbol{C}_{\boldsymbol{o-}}\boldsymbol{C}_{\boldsymbol{e}}\boldsymbol{)}}{\boldsymbol{m}}\boldsymbol{V}$ (1)

Where C_0_ (mg/L) is the initial BTX concentrations, C_e_ (mg/L) is the concentration at equilibrium, m (g) is the mass of MNP-OA nanocomposites, and V (L) is the volume of solution.

**S2: Adsorption Studies Dynamics**

For the adsorption dynamic studies, the effect of pH and adsorbent dosage were further carried out, most especially pH solution significantly influences the adsorption mechanism of BTX removal [S1]. To study the effect of solution pH, 0.1 g of MNP and MNP-OA (At optimized RSM model conditions) were added to each flask containing 50 mL solution of BTX with initial concentration of 100 mg/L set at the pH range (3-12). To study the effect of adsorbent dose, various amounts of adsorbent ranging from 0.05 to 1.5 g were added to each flask containing 50 mL solution of BTX with initial concentration of 100 mg/L set at pH 7 for MNP and pH 8 for MNP-OA.

**S2.1: Effect of solution pH**

The solution pH effect on the adsorption of BTX onto MNP, and MNP-OA was studied at the solution pH 3, 4, 5, 6, 7, 8, 9 10, 11 and 12 respectively, and thus presented in **Fig. S1A and 1B,** respectively. From the plots, it was observed that the adsorption of BTX was pH dependant. The amount of BTX adsorbed onto MNP was observed to increase with an increase in solution pH from pH 3 to pH 7, then reduced as the pH increased from pH 7 to pH 12. On the other hand, the adsorption capacity of BTX onto MNP-OA increased with an increase in solution pH from pH 3 to pH 8 then steadily reduced as solution pH increased to pH 12. The adsorption mechanism of BTX onto magnetite based adsorbents can be explained in relation to acid–base chemistry and by considering the pH_pzc_ of the adsorbents. pH_pzc_ of magnetite was 6.9 and that om MNP-OA was 6.4 [S2, S3]. The adsorbent surface is positively charged at pH lower than pH_pzc_ and negatively charged at pH above pH_pzc_. At low solution pH, there are high amount of H^+^ in solution which may protonate the oxygen group on MNP and the oxygenated functional groups on the MNP-OA composite to form positive charges, $Fe-OH_{2}^{+}$and $R-OH_{2}^{+}$. Low adsorption capacity will be observed at low solution pH as the protonated groups cannot bind with benzene, toluene and xylene. Electrons have been reported to be donated from the phenyl ring of the benzene, toluene and xylene to the surface of iron atoms, and in turn providing electron density to unoccupied 3d orbitals of iron cations. [S4, S5]. At higher solution pH, where the surface of both adsorbents are negatively charged, a reduction in the BTX uptake was observed due repulsion of the negative charge on MNP and MNP-OA with the π electrons on the BTX rings.

**S2.2: Effect of adsorbent dose**

The effect of mass of MNP and MNP-OA composite on the uptake of BTX from aqueous solution is displayed in **Fig S2 A to D.** It was observed that the increase in MNP and MNP-OA mass from 0.05 to 1.5 g led to a reduction in adsorption capacity. The adsorption capacity of MNP reduced from 59.51 to 2.52 mg/g for benzene, 63.0 to 3.84 mg/g for toluene and from 69.6 to 8.00 mg/g for xylene while that of MNP-OA composite reduced from 77.1 to 2.84 mg/g for benzene, from 80.49 to 5.2 mg/g for toluene and from 85.70 to 10.2 mg/g for xylene. However, the percentage adsorption of the BTX showed a reverse trend. The percentage removal increased from 56.48 to 82.82 % for benzene, 63.54 to 83.87 % for toluene and from 69.54 to 85.02 % for xylene while that of MNP-OA composite increased from 77.45 to 85.95 % for benzene, from 79.34 to 87.24 % for toluene and from 79.84 to 88.14 % for xylene. The percentage removal was observed to increase with increasing adsorbent dose and became almost constant on reaching mass of adsorbent of 0.5g. This may be due to the increase in the external surface or accessible adsorption active sites on the MNP and MNP-OA surface as the mass of adsorbent increases [S6]. Similar results were reported by Anjum et al. (2019) on BTX removal using multiwall carbon nanotubes [1].

Moreover, the significant impact of volume of oleic acid (VOA) from the RSM model on MNP-OA composite, critically influenced its enhanced adsorptive removal in comparison to MNP, judging from the adsorption parametric studies discussed above. Clearly, this was achieved through uniform dispersion, reduced particle size, and more active chemical functionalities [S7] on the surface of MNP-OA, thus contributing to a strong chemical reaction with the adsorbate molecules, resulting in enhancement of BTX adsorption. Also, a synergistic electrostatic interaction by the electron clouds (X > T > B) is evident (physical adsorption), thus emanating from interaction of MNP-OA, and BTX [S8].


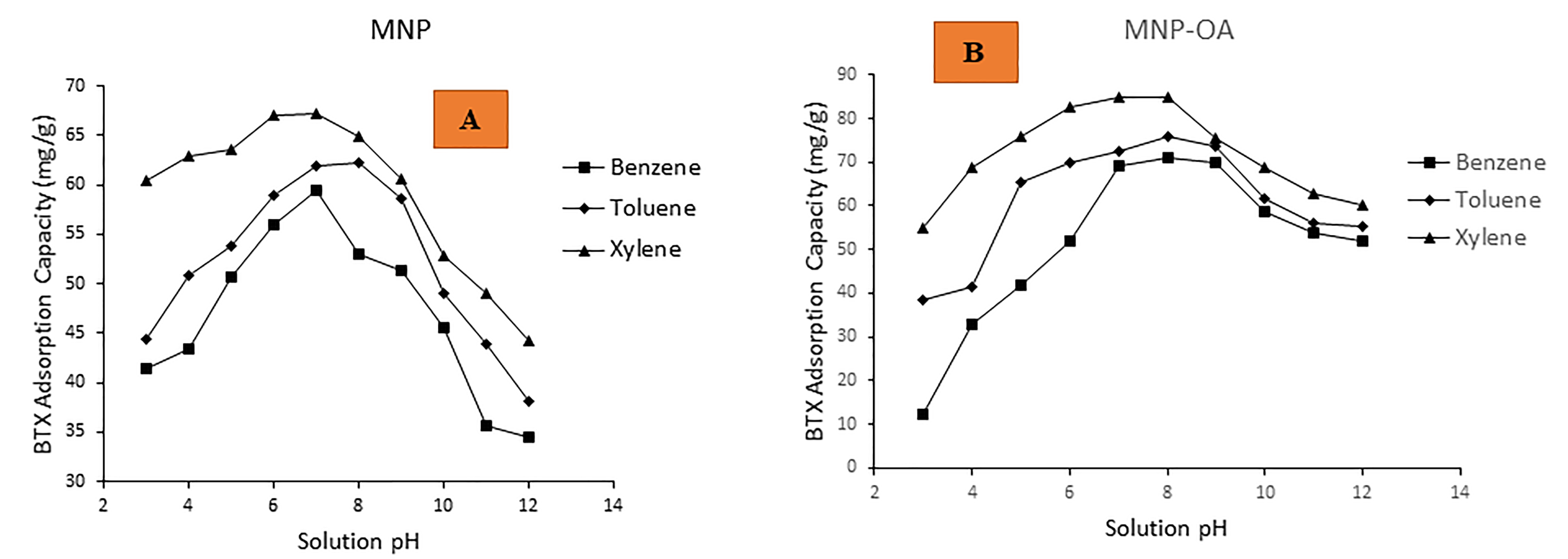


**Fig. S1**: Effect of solution pH on the uptake of BTX from aqueous solution by (a) MNP and (b) MNP-OA composite.


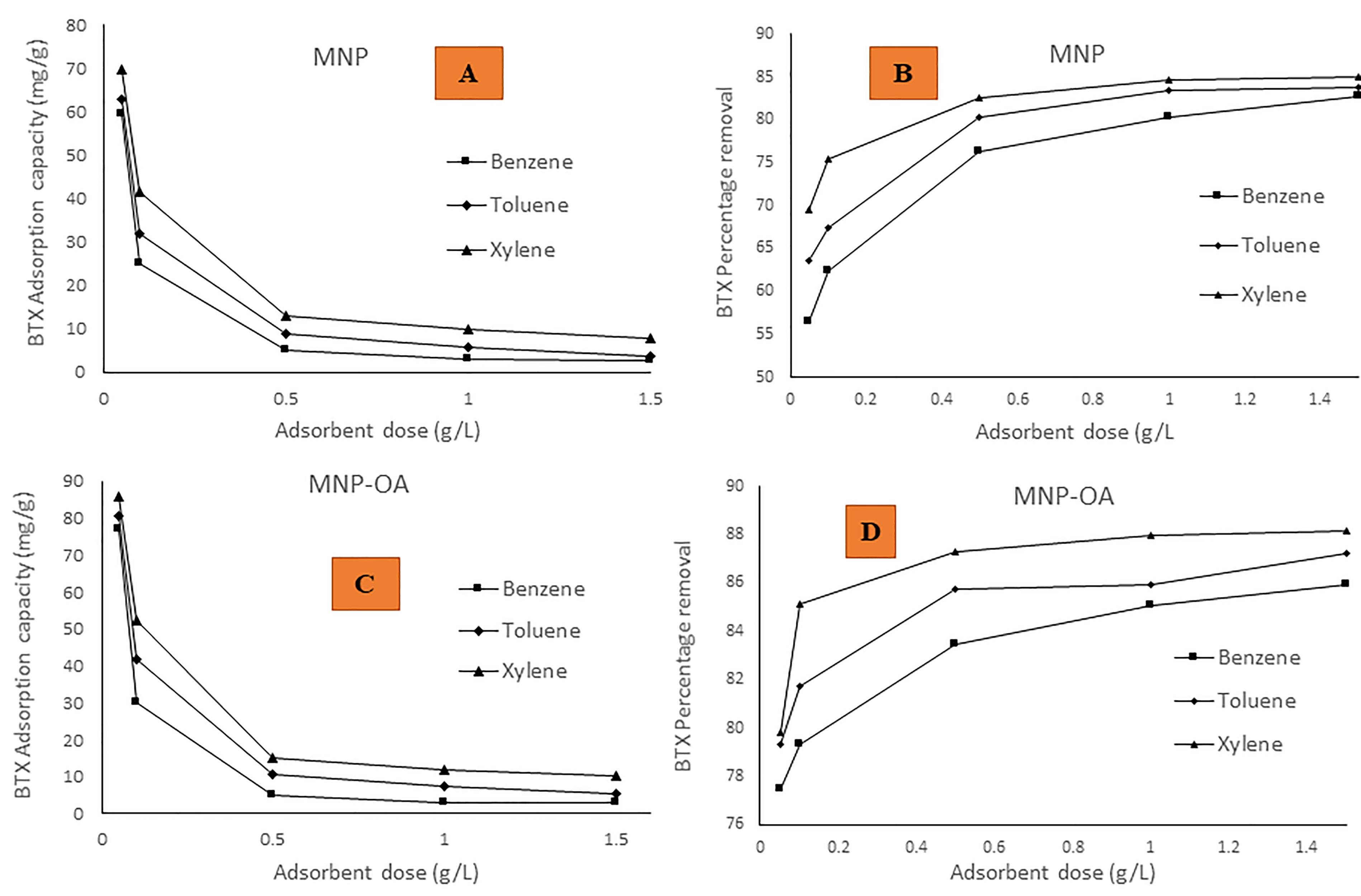


**Fig. S2**: The effect of Adsorbent dosage on the uptake of BTX onto (a, b) MNP and (c, d) MNP-OA composite.

**Table S1 Analysis of variance for % Fe content**

| **Source** | **Sum of Squares** | **df** | **Mean Square** | **F-value** | **p-value** |  |
| --- | --- | --- | --- | --- | --- | --- |
| **Model** | 5002,04 | 20 | 250,10 | 96,81 | < 0.0001 | significant |
| A-Fe^3+/^Fe^2+^ | 172,58 | 1 | 172,58 | 66,80 | < 0.0001 |  |
| B-Microwave Power | 94,22 | 1 | 94,22 | 36,47 | < 0.0001 |  |
| D-Time | 25,77 | 1 | 25,77 | 9,97 | 0,0037 |  |
| E-Volume of Oleic acid | 1511,56 | 1 | 1511,56 | 585,08 | < 0.0001 |  |
| AD | 825,20 | 1 | 825,20 | 319,41 | < 0.0001 |  |
| AE | 29,45 | 1 | 29,45 | 11,40 | 0,0021 |  |
| BC | 61,33 | 1 | 61,33 | 23,74 | < 0.0001 |  |
| BD | 38,06 | 1 | 38,06 | 14,73 | 0,0006 |  |
| BE | 27,20 | 1 | 27,20 | 10,53 | 0,0030 |  |
| CD | 115,90 | 1 | 115,90 | 44,86 | < 0.0001 |  |
| CE | 233,82 | 1 | 233,82 | 90,50 | < 0.0001 |  |
| DE | 233,82 | 1 | 233,82 | 90,50 | < 0.0001 |  |
| B² | 44,49 | 1 | 44,49 | 17,22 | 0,0003 |  |
| D² | 230,74 | 1 | 230,74 | 89,31 | < 0.0001 |  |
| E² | 54,84 | 1 | 54,84 | 21,23 | < 0.0001 |  |
| **Residual** | 74,92 | 29 | 2,58 |  |  |  |
| Lack of Fit | 47,94 | 22 | 2,18 | 0,5654 | 0,8553 | not significant |
| Pure Error | 26,98 | 7 | 3,85 |  |  |  |
| **Cor Total** | 5076,96 | 49 |  |  |  |  |

**Table S2 Analysis of variance for benzene adsorption capacity**

| **Source** | **Sum of Squares** | **df** | **Mean Square** | **F-value** | **p-value** |  |
| --- | --- | --- | --- | --- | --- | --- |
| **Model** | 12615,72 | 20 | 630,79 | 85,36 | < 0.0001 | significant |
| B-Microwave Power | 593,84 | 1 | 593,84 | 80,36 | < 0.0001 |  |
| C-Volume of NH_4_OH | 666,71 | 1 | 666,71 | 90,23 | < 0.0001 |  |
| D-Time | 82,76 | 1 | 82,76 | 11,20 | 0,0023 |  |
| E-Volume of Oleic acid | 492,37 | 1 | 492,37 | 66,63 | < 0.0001 |  |
| AB | 335,94 | 1 | 335,94 | 45,46 | < 0.0001 |  |
| AC | 49,59 | 1 | 49,59 | 6,71 | 0,0148 |  |
| AE | 153,68 | 1 | 153,68 | 20,80 | < 0.0001 |  |
| BC | 805,34 | 1 | 805,34 | 108,99 | < 0.0001 |  |
| BD | 326,69 | 1 | 326,69 | 44,21 | < 0.0001 |  |
| CD | 226,70 | 1 | 226,70 | 30,68 | < 0.0001 |  |
| CE | 478,22 | 1 | 478,22 | 64,72 | < 0.0001 |  |
| DE | 172,94 | 1 | 172,94 | 23,40 | < 0.0001 |  |
| A² | 383,58 | 1 | 383,58 | 51,91 | < 0.0001 |  |
| B² | 808,30 | 1 | 808,30 | 109,39 | < 0.0001 |  |
| C² | 1888,10 | 1 | 1888,10 | 255,52 | < 0.0001 |  |
| D² | 729,36 | 1 | 729,36 | 98,70 | < 0.0001 |  |
| E² | 674,98 | 1 | 674,98 | 91,34 | < 0.0001 |  |
| **Residual** | 214,29 | 29 | 7,39 |  |  |  |
| Lack of Fit | 182,66 | 22 | 8,30 | 1,84 | 0,2083 | not significant |
| Pure Error | 31,64 | 7 | 4,52 |  |  |  |
| **Cor Total** | 12830,01 | 49 |  |  |  |  |

**Table S3 Analysis of variance for toluene adsorption capacity**

| **Source** | **Sum of Squares** | **df** | **Mean Square** | **F-value** | **p-value** |  |
| --- | --- | --- | --- | --- | --- | --- |
| **Model** | 13187,98 | 20 | 659,40 | 55,63 | < 0.0001 | significant |
| B-Microwave Power | 467,24 | 1 | 467,24 | 39,42 | < 0.0001 |  |
| C-Volume of NH_4_OH | 708,62 | 1 | 708,62 | 59,78 | < 0.0001 |  |
| D-Time | 107,90 | 1 | 107,90 | 9,10 | 0,0053 |  |
| E-Volume of Oleic acid | 589,89 | 1 | 589,89 | 49,76 | < 0.0001 |  |
| AB | 301,78 | 1 | 301,78 | 25,46 | < 0.0001 |  |
| AE | 203,26 | 1 | 203,26 | 17,15 | 0,0003 |  |
| BC | 1095,71 | 1 | 1095,71 | 92,44 | < 0.0001 |  |
| BD | 368,90 | 1 | 368,90 | 31,12 | < 0.0001 |  |
| CD | 188,42 | 1 | 188,42 | 15,90 | 0,0004 |  |
| CE | 387,18 | 1 | 387,18 | 32,66 | < 0.0001 |  |
| DE | 122,50 | 1 | 122,50 | 10,33 | 0,0032 |  |
| A² | 402,46 | 1 | 402,46 | 33,95 | < 0.0001 |  |
| B² | 760,39 | 1 | 760,39 | 64,15 | < 0.0001 |  |
| C² | 1981,74 | 1 | 1981,74 | 167,18 | < 0.0001 |  |
| D² | 657,24 | 1 | 657,24 | 55,45 | < 0.0001 |  |
| E² | 648,61 | 1 | 648,61 | 54,72 | < 0.0001 |  |
| **Residual** | 343,76 | 29 | 11,85 |  |  |  |
| Lack of Fit | 310,78 | 22 | 14,13 | 3,00 | 0,0700 | not significant |
| Pure Error | 32,98 | 7 | 4,71 |  |  |  |
| **Cor Total** | 13531,74 | 49 |  |  |  |  |

**Table S4 Analysis of variance for xylene adsorption capacity**

| **Source** | **Sum of Squares** | **df** | **Mean Square** | **F-value** | **p-value** |  |
| --- | --- | --- | --- | --- | --- | --- |
| **Model** | 14804,53 | 20 | 740,23 | 61,42 | < 0.0001 | significant |
| B-Microwave Power | 613,84 | 1 | 613,84 | 50,94 | < 0.0001 |  |
| C-Volume of NH_4_OH | 697,37 | 1 | 697,37 | 57,87 | < 0.0001 |  |
| D-Time | 78,51 | 1 | 78,51 | 6,51 | 0,0162 |  |
| E-Volume of Oleic acid | 450,12 | 1 | 450,12 | 37,35 | < 0.0001 |  |
| AB | 334,37 | 1 | 334,37 | 27,75 | < 0.0001 |  |
| AC | 62,01 | 1 | 62,01 | 5,15 | 0,0309 |  |
| AE | 155,17 | 1 | 155,17 | 12,88 | 0,0012 |  |
| BC | 810,59 | 1 | 810,59 | 67,26 | < 0.0001 |  |
| BD | 310,75 | 1 | 310,75 | 25,79 | < 0.0001 |  |
| CD | 228,83 | 1 | 228,83 | 18,99 | 0,0002 |  |
| CE | 527,10 | 1 | 527,10 | 43,74 | < 0.0001 |  |
| DE | 164,38 | 1 | 164,38 | 13,64 | 0,0009 |  |
| A² | 127,65 | 1 | 127,65 | 10,59 | 0,0029 |  |
| B² | 1414,15 | 1 | 1414,15 | 117,35 | < 0.0001 |  |
| C² | 5409,03 | 1 | 5409,03 | 448,84 | < 0.0001 |  |
| D² | 329,53 | 1 | 329,53 | 27,34 | < 0.0001 |  |
| E² | 1170,95 | 1 | 1170,95 | 97,16 | < 0.0001 |  |
| **Residual** | 349,48 | 29 | 12,05 |  |  |  |
| Lack of Fit | 317,42 | 22 | 14,43 | 3,15 | 0,0619 | not significant |
| Pure Error | 32,06 | 7 | 4,58 |  |  |  |
| **Cor Total** | 15154,01 | 49 |  |  |  |  |

**References**

1. Anjum, H., Johari, K., Gnanasundaram, N., Appusamy, A., and Thanabalan, M. Investigation of green functionalization of multiwall carbon nanotubes and its application in adsorption of benzene, toluene & p-xylene from aqueous solution. J. Cleaner Prod. **221**, 323-338, (2019).

2. Ouma, L., and Ofomaja, A.J.R.a. Probing the interaction effects of metal ions in Mn x Fe (3− x) O 4 on arsenite oxidation and adsorption. *RSC Adv* **10**, 2812-2822, (2020)

3. Masuku, M., Ouma, L., and Pholosi, A. Microwave assisted synthesis of oleic acid modified magnetite nanoparticles for benzene adsorption. *Environ. Nanotechnol. Manag* **15**, 100429, (2021).

4. Joseph, Y., Wühn, M., Niklewski, A., Ranke, W., Weiss, W., Wöll, C., and Schlögl, R. Interaction of ethylbenzene and styrene with iron oxide model catalyst films at low coverages: A NEXAFS study. *Phys. Chem. Chem. Phys* **2**, 5314-5319, (2000).

5. Sasaki, T., and Tanaka, S. Adsorption behavior of some aromatic compounds on hydrophobic magnetite for magnetic separation. *J. Hazard. Mater* **196**, 327-334 (2011).

6. Pholosi, A., Naidoo, E., and Ofomaja, A. Sequestration of As (III) pollutant from water using chemically activated pine cone biomass: evaluation of interaction and mechanism. *Int. J. Environ. Sci* **16**, 6907-6920 (2019).

7. Kutluay, S. Excellent adsorptive performance of novel magnetic nano-adsorbent functionalized with 8-hydroxyquinoline-5-sulfonic acid for the removal of volatile organic compounds (BTX) vapors. *Fuel* **287**, 119691, (2021).

8. Ece, M.Ş., and Kutluay, S. Comparative and competitive adsorption of gaseous toluene, ethylbenzene, and xylene onto natural cellulose-modified Fe_3_O_4_ nanoparticles. *J. Environ. Chem. Eng* **10**, 107389, (2022).
